# Secondary-Atom-Doping Enables Robust Fe–N–C Single-Atom Catalysts with Enhanced Oxygen Reduction Reaction

**DOI:** 10.1007/s40820-020-00502-5

**Published:** 2020-08-12

**Authors:** Xin Luo, Xiaoqian Wei, Hengjia Wang, Wenling Gu, Takuma Kaneko, Yusuke Yoshida, Xiao Zhao, Chengzhou Zhu

**Affiliations:** 1 Key Laboratory of Pesticide and Chemical Biology of Ministry of Education, International Joint Research Center for Intelligent Biosensing Technology and Health, College of Chemistry, Central China Normal University, Wuhan, 430079 People’s Republic of China; 2 Innovation Research Center for Fuel Cells, The University of Electro-Communications, Chofugaoka, Chofu, Tokyo 182-8585 Japan

**Keywords:** Single-atom catalysts, Fe–N–C catalysts, Doping, Porous nanostructures, Oxygen reduction reaction

## Abstract

Single-atom catalysts (SACs) with nitrogen-coordinated nonprecious metal sites have exhibited inimitable advantages in electrocatalysis. However, a large room for improving their activity and durability remains. Herein, we construct atomically dispersed Fe sites in N-doped carbon supports by secondary-atom-doped strategy. Upon the secondary doping, the density and coordination environment of active sites can be efficiently tuned, enabling the simultaneous improvement in the number and reactivity of the active site. Besides, structure optimizations in terms of the enlarged surface area and improved hydrophilicity can be achieved simultaneously. Due to the beneficial microstructure and abundant highly active FeN_5_ moieties resulting from the secondary doping, the resultant catalyst exhibits an admirable half-wave potential of 0.81 V versus 0.83 V for Pt/C and much better stability than Pt/C in acidic media. This work would offer a general strategy for the design and preparation of highly active SACs for electrochemical energy devices.

**Electronic supplementary material:**

The online version of this article (10.1007/s40820-020-00502-5) contains supplementary material, which is available to authorized users.

## Highlights

- Secondary-atom-doped strategy was proposed to synthesize single-atom electrocatalyst.
- The increase in both the density of active sites and their intrinsic activity was achieved simultaneously.
- The resultant single-atom catalyst shows outstanding ORR activity in acidic media.

**Electronic supplementary material:**

The online version of this article (10.1007/s40820-020-00502-5) contains supplementary material, which is available to authorized users.

## Graphical abstract

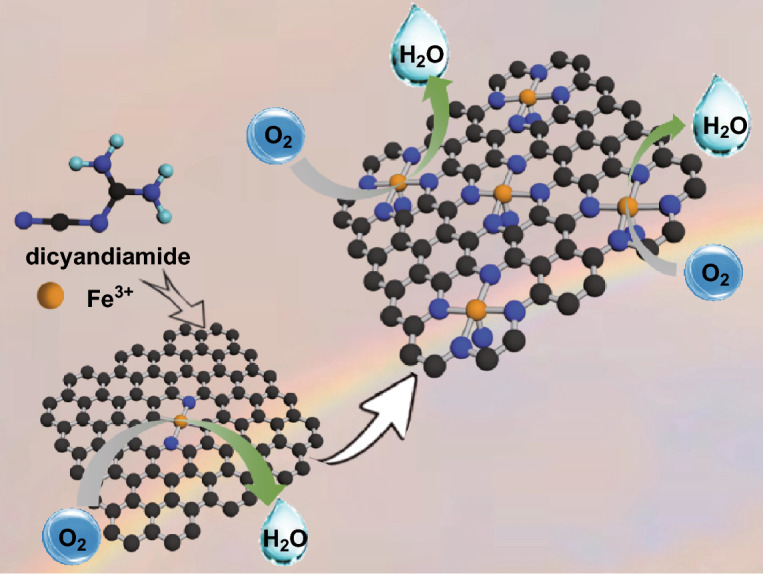

## Introduction

Tremendous efforts have been devoted to the exploration of environment-friendly and sustainable energy conversion [1–4]. Among the various options of devices, the sustainable and clean fuel cells involving electrochemical oxygen reduction reaction (ORR) have been regarded as promising energy conversion applications [5–8]. So far, Pt-based catalysts with superior activity are promising electrocatalysts for the ORR [9–12]. However, their inhibitive cost, scarcity and instability call for nonprecious metal catalysts (NPMCs) that have the advantages of natural abundance and low cost [13–17]. In this context, single-atom catalysts (SACs) with nitrogen-coordinated transition metal sites and maximized atom-utilization efficiency, for example, M–N–C (M = Fe, Co, Ni, etc.), are emerging as a promising candidate for the ORR [18–22]. The tailored electronic structure and coordination environments of SACs bring new possibilities for improving ORR performance and fundamental understanding of active centers due to their structural simplicity [23–25]. An annealing process has been demonstrated for the effective formation of active metal centers, which however causes the difficulty in controlling the microstructure/porosity and coordination environment of the SACs [26–28]. Furthermore, to achieve atomic dispersion of metal atoms, SACs usually have a very low density of metal sites that limits catalytic performance in practical devices.

To overcome these limitations of SACs, several strategies have been developed to improve catalytic activity [29–36]. Introducing heteroatoms (N, B, P, and S, etc.) or defect in materials can change the coordination environments of metal sites and thus improve the intrinsic activity of active site, which have been evidenced the effectiveness of enhancing the ORR performance of SACs [30, 37–39]. In addition to optimizing their intrinsic activity of active sites, tuning the number of active sites is also important. Structure design, such as size or morphology control, can improve the exposed area and density of active sites, whereas this strategy is difficult in tuning the coordination environment and intrinsic activity of metal centers [37, 40, 41]. Despite a significant advance in developing SACs, the methods of enhancing concentration and regulating the coordination environment of metal sites simultaneously are still rarely reported. Besides the above, engineering microstructure and hydrophobic/hydrophilic properties of catalysts can significantly realize a high ORR performance [42].

Herein, we reported a secondary-atom-doped strategy to synthesize atomical Fe sites dispersed in carbon support catalysts (Fe–N–C/FeN). Through combining dual-template synthetic strategy involving silica sols as hard templates and ZnCl_2_ as a pore-forming agent, Fe–N–C was first prepared by the pyrolysis and acid leaching. Then, the secondary-atom-doping involving the second thermal activation in the presence of metal salt and nitrogen precursor was carried out to synthesize Fe–N–C/FeN. The final catalyst exhibits an enlarged specific surface area and improved hydrophilicity, which could offer more active sites and make active sites accessible. Significantly, the secondary-atom-doping process enables to simultaneously tune the density and the coordination environment of the single atoms, achieving the goal of substantially boosting catalytic activity. As expected, the as-synthesized Fe–N–C/FeN possesses a remarkable electrocatalytic activity in acidic solution with the remarkable onset potential (*E*_onset_, 0.85 V) and half-wave potential (*E*_1/2_, 0.81 V) and high stability with scarcely any decay in *E*_1/2_ after 30,000 cycles, which exhibit comparable activity but superior stability relative to commercial Pt/C.

## Experimental Section

### Physical Characterizations

The morphology of the as-prepared samples was studied by transmission electron microscope (TEM, JEM-2100 HR), high-angle annular dark-field scanning transmission electron microscopy and elemental distribution mappings (HAADF-STEM and EDS mappings, a JEOL 2100F electron microscope). The phase analysis was measured by Raman spectrometer (J-Y T64000), the X-ray diffraction (XRD, X’Pert PRO MRD) and the X-ray photoelectron spectrometer (XPS, PHI-5702). Brunauer–Emmett–Teller (BET) operated on ASAP 2020. The synchrotron-based X-ray absorption spectroscopy (XAS) data were processed by IFEFFIT software.

### Materials Preparation

2 g D-glucosamine hydrochloride, ZnCl_2_ and FeCl_3_ with molar ratios (10: 2: 1) were dissolved in 20 mL colloidal silica suspension (2 g SiO_2_) with stirring for 10 min. After freeze-dried, the as-prepared samples were heated to 900 °C for 2 h in the atmosphere of N_2_ at 5 °C min^−1^ (ramping rate). The as-obtained material was treated with HF (10 wt%) etching for 12 h and the following drying. The black product is labeled as Fe–N–C.

Afterward, 0.4 g Fe–N–C, 26.3 mg FeCl_3_ and 0.4 g dicyandiamide were mixed with 20 mL of water and 20 mL of isopropanol by 2 h ultrasound and 5 h stirred, then dried at 60 °C. The precursor was annealed at 900 °C with 3 °C min^−1^ (ramping rate) for 1 h under the atmosphere of N_2_. After the secondary-atom-doping process, the final catalyst was called as Fe–N–C/FeN. For comparison, Fe–N–C/control and Fe–N–C/N catalysts were synthesized through similar procedures with no additional Fe and N source and only additional N source in the second step, respectively.

### Electrochemical Experiments

The electrochemical activities were conducted by using a CHI-660 electrochemical analyzer at room temperature. 2 mg of catalysts was dispersed in 5 μL of Nafion (5 wt%), 100 μL of isopropyl alcohol and 400 μL of deionized water by ultrasonic treatment for 30 min to obtain uniform electrocatalyst inks. Then such inks were cast on the surface of rotating disk electrode (RDE) and rotating ring-disk electrode (RRDE) in O_2_-saturated 0.1 M HClO_4_ solution with a loading of 1.0 mg cm^−2^. The electrochemical active surface area (ECSA) was tested by cyclic voltammetry. Electrochemical impedance spectroscopy (EIS) was measured under AC voltage amplitude of 5 mV and DC voltage based at a given potential at 10 mA cm^−2^ from 100 kHz to 0.05 Hz. Tafel slopes were calculated as follows:

1 $$\eta = a + b\log (j/j_{0} )$$

where *η*, *b*, *j*, and *j*_0_ are the overpotential, the Tafel slope, the current density, and the exchange current density, respectively. The long-term stability and methanol poisoning experiments were investigated by chronoamperometric measurements. The accelerated durability test (ADT) was investigated by continuous potential cycling between 0.7 and 1 V.

The Koutecky–Levich (K–L) equation was as follows:

2 $$\frac{1}{J} = \frac{1}{{J_{\text{L}} }} + \frac{1}{{J_{\text{K}} }} = \frac{1}{{Bw^{1/2} }} + \frac{1}{{J_{\text{K}} }}$$

3 $$B = 0.2nFC_{0} D_{0}^{2/3} V^{ - 1/6}$$

where *J*, *J*_K_, and *J*_L_ are the measured current density, kinetic current density and the diffusion-limiting current density, respectively; *ω* is the angular velocity; *n* and *F* is the electron transfer number and the Faraday constant (96 485 C mol^−1^); *C*_0_ and *D*_0_ are the bulk concentration of O_2_ (1.2 × 10^−6^ mol cm^−3^) and the diffusion coefficient of O_2_ (1.9 × 10^−5^ cm^2^ s^−1^); *V* is the kinematic viscosity of the electrolyte (0.01 cm^2^ s^−1^).

The specific kinetic current density can be determined according to the K-L equation:

4 $$J_{\text{K}} = \frac{{J_{\text{L}} *J}}{{J_{\text{L}} - J}}$$

The H_2_O_2_ yield (*y*) and *n* are computed using Eqs. (5) and (6):

5 $${\text{H}}_{2} {\text{O}}_{2} \left( \% \right) = \frac{{200I_{\text{r}} /N}}{{I_{\text{r}} /N + I_{\text{d}} }}$$

6 $$n = \frac{{4I_{\text{d}} }}{{I_{\text{r}} /N + I_{\text{d}} }}$$

where *I*_r_ and *I*_d_ are ring and disk current; the current collection efficiency of the Pt ring (N) is 0.37.

The specific value *S*_a_ can be calculated by the related Eqs. (7) and (8):

7 $$C = I/vm$$

8 $$S_{\text{a}} = C/C_{\text{GC}}$$

where *v* is a given scan rate; *m* is mass of catalyst deposited on the electrode; C and *C*_GC_ are the gravimetric double-layer capacitance (F g^−1^) and the double-layer capacitance (F m^−2^) of the electrode surface, respectively.

## Results and Discussion

### Materials Characterizations

**Fig. 1:**
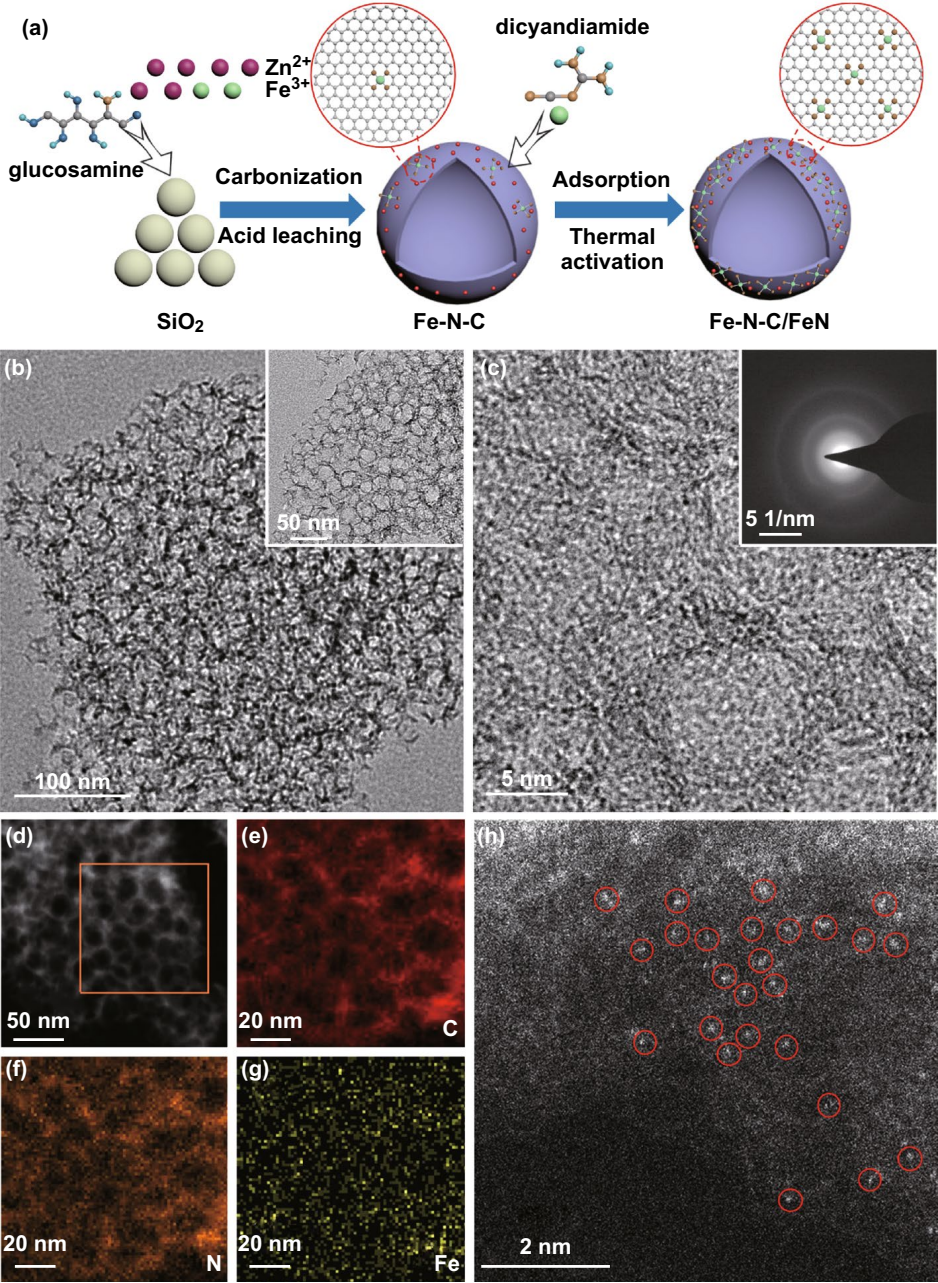
**a** Schematic illustration of the synthesis route. **b** TEM and **c** HRTEM images of Fe–N–C/FeN. Inset in **c**: SAED pattern of Fe-N-C/FeN. **d-g** HAADF-STEM and relative elemental mapping images. **h** AC-STEM image of Fe-N-C/FeN

The Fe–N–C/FeN catalyst was successfully synthesized via a two-step pyrolysis process (Fig. 1a). First, Fe–N–C was synthesized by using a dual-template strategy according to our previous report [43]. Typically, silica sol suspension, ZnCl_2_, FeCl_3_, and glucosamine were mixed to freezing dry and followed by the pyrolysis and acid leaching. Finally, Fe–N–C/FeN were obtained through the pyrolysis of the mixture of FeCl_3_ and dicyandiamide in the presence of the as-prepared Fe–N–C. As revealed in Fig. 1b, TEM image of Fe–N–C/FeN shows that the catalyst was composed of hierarchically spherical pores with the size of about 20 nm, which inherits the morphology of silica nanospheres. HRTEM image in Fig. 1c shows that the thickness of graphene layers of Fe–N–C/FeN is around 2 nm and no nanoparticles or clusters can be observed. As expected, Fe–N–C/N, Fe–N–C/control, and Fe–N–C show similar morphology to Fe–N–C/FeN, revealing that the secondary-doped atoms did not affect the original morphology (Fig. S1). The ring-like selected area electron diffraction (SAED) pattern verifies the low crystallinity of the sample, demonstrating the disordered stack of graphene layers (inset in Fig. 1c). As revealed in Fig. 1d-g, HAADF-STEM and corresponding EDS mappings were conducted, where Fe, C, and N elements are uniformly dispersed. The aberration-corrected scanning transmission electron microscope (AC-STEM) image reveals that Fe single atoms with brighter spots marked are facilely observed over the hierarchically porous structures (Fig. 1h).

**Fig. 2:**
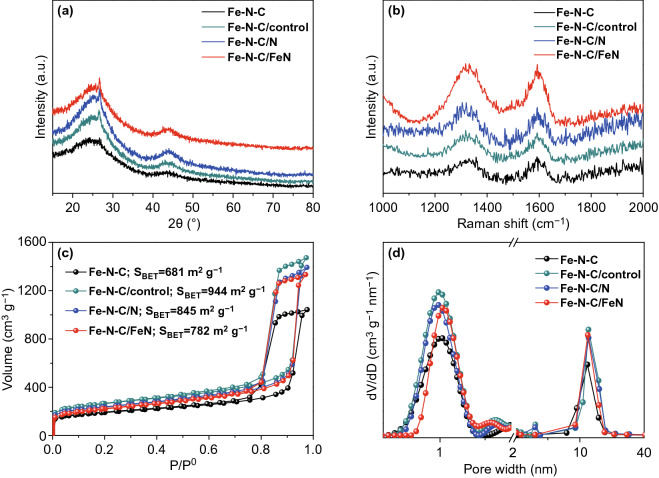
**a** XRD patterns, **b** Raman spectra, **c** nitrogen adsorption–desorption isotherm curves and **d** the corresponding pore size distribution curves of the resultant catalysts

As shown in XRD patterns, the catalysts show two distinct peaks at 25° and 43° (Fig. 2a), which are identified as a certain degree of graphitized carbon structures [37]. Except for the two graphitized carbon peaks, no other peaks ascribed to Fe species were observed, indicating the single-atom features of the resultant carbon structures. Fe–N–C/FeN and Fe–N–C/N had a similar crystal structure to Fe–N–C, further suggesting the secondary-doped Fe or N atoms did not destroy the overall structure of supports. Furthermore, the obtained catalysts were characterized by Raman spectra. Two sharp characteristic peaks of these catalysts in accordance with the characteristic D band at 1350 cm^−1^ and G band at 1580 cm^−1^ were assigned to the lattice defects and *sp*^2^-hybridized carbon, respectively (Fig. 2b). The intensity ratio of D to G band (*I*_D_/*I*_G_) manifests the degree of graphitization of carbon materials [44]. Among the as-synthesized catalysts, the *I*_D_/*I*_G_ (1.25) of Fe–N–C/FeN is higher than those of Fe–N–C (0.86), Fe–N–C/control (1.05) and Fe–N–C/N (1.06). This result manifests that the introduction of secondary-doped atoms can be capable of generating more abundant defects, benefiting the exposure of active sites [45]. The N_2_ adsorption–desorption curves of catalysts exhibit a type-IV isotherm, revealing the catalysts possess micropores and mesopores structure (Fig. 2c). It was observed that the BET surface area of the resultant catalysts increased in the following order: Fe–N–C (681 m^2^ g^−1^) < Fe–N–C/FeN (782 m^2^ g^−1^) < Fe–N–C/N (845 m^2^ g^−1^) < Fe–N–C/control (944 m^2^ g^−1^). BET surface areas of all catalysts subjected to second thermal treatment are larger than that of Fe–N–C, manifesting that the second thermal activation can effectively improve the surface area of materials. Further observation reveals that the secondary-doped N source leads to the decrease in the surface area of materials relative to Fe–N–C/control, and the surface area further decreases after secondary-doped Fe and N source. Based on the pore size distribution (Fig. 2d), the mesopore distribution of Fe–N–C/FeN is similar to that of Fe–N–C. As for the micropore distribution, Fe–N–C only possesses one micropore located at 1.1 nm, which attributes to the evaporation of Zn at high temperatures [46]. However, Fe–N–C/control, Fe–N–C/N, and Fe–N–C/FeN display an extra micropore located at 1.5 nm, which may attribute to the second thermal activation. Compared with Fe–N–C/control, Fe–N–C/N and Fe–N–C/FeN exhibited the decrease in micropores and mesopores volume, which may attribute to that Fe and N sources are stabilized in the micropores and mesopores via possible coordination (Table S1) [47]. These results further confirmed that Fe–N–C/FeN possesses the hierarchically porous structure and high surface area, which could offer ample space to improve the density of active sites and promote the transport of proton [46].

**Fig. 3:**
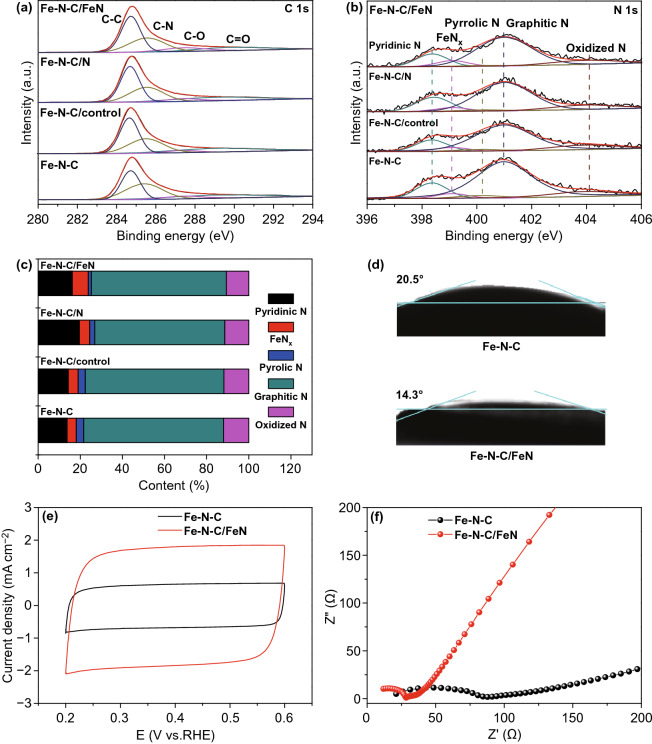
**a** C 1s, **b** N 1s XPS spectra, and **c** corresponding contents of different N species of the resultant catalysts. **d** Water contact angle of Fe–N–C and Fe–N–C/FeN. **e** Cyclic voltammetry curves in N_2_-saturated HClO_4_ solution (sweep rate: 10 mV s^−1^). **f** EIS curves of Fe–N–C and Fe–N–C/FeN

To analyze the effect of secondary-atom-doping on the compositions and chemical states, XPS was performed. As expected, the C content of Fe–N–C/FeN increases in comparison with Fe–N–C, while the O and N content in the catalysts decreases, which may be attributed to the repairs of carbon supports at the second pyrolysis process. (Table S2) [47]. The high-resolution C 1s spectrum of Fe–N–C/FeN (Fig. 3a) exhibits four different C species, corresponding to C–C (289.8 eV), C–N (287.4 eV), C–O (285.5 eV), and C=O group (284.7 eV), respectively [48, 49]. The C–C species content increases after the second thermal activation, which may be attributed to the increase of C content (Table S3). As for the high-resolution N 1s spectrum of Fe–N–C/FeN (Fig. 3b), it could be deconvoluted with five peaks at 398.3 (pyridinic-N), 399.1 (FeN_*x*_), 400.1 (pyrrolic-N), 401.0 (graphitic-N), and 403.9 eV (oxidized N), respectively [50]. Although the N content decreased, the percentage of FeN_*x*_ increased from 4.3% (Fe–N–C) to 7.6% (Fe–N–C/FeN) during the second thermal activation in Fig. 3c and Table S4. In addition to forming additional FeN_*x*_, the secondary-doped N atoms also benefit the transformation of pyrrolic-N into pyridinic-N, which can promote the electrochemical ORR activity in acidic media [51]. Inductively coupled plasma-mass spectrometry (ICP-MS) test was performed to confirm the content of Fe single atoms (Table S5). The Fe content of Fe–N–C/FeN (0.38 wt%) is twice higher than that of Fe–N–C, indicating additional Fe atoms was successfully doped in initial material. Thanks to the advantageous structure and composition, the hydrophilicity has been significantly improved after the second doping by the contact angle (CA) measurement. As shown in Fig. 3d, the contact angle (14.3°) of Fe–N–C/FeN was smaller than that of Fe–N–C (20.5°), which is proved to facilitate the accessibility of active sites to the electrolyte [42]. ECSA was investigated to further estimate the intrinsic activity of the active sites (Fig. 3e). The ECSA of Fe–N–C/FeN (910 cm^2^ g^−1^) is about 3 times larger than that of Fe–N–C, manifesting that the secondary-atom dopant in Fe–N–C/FeN leads to the higher intrinsic activity. Besides, the improved electrical conductivity of Fe–N–C/FeN was characterized by EIS (Fig. 3f). The charge transfer resistance (*R*_ct_) between electrolyte and catalyst is in connection with the semicircle region of Nyquist plots. Compared with Fe–N–C, Fe–N–C/FeN possesses a small *R*_ct_, indicating that the optimized porous nanostructures caused by secondary-atom-doping are also beneficial to electron transport.

**Fig. 4:**
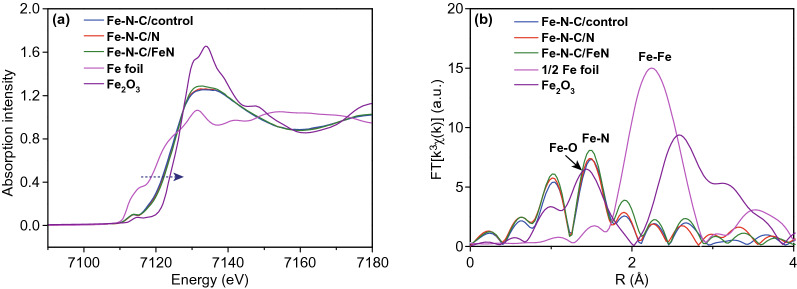
**a** K-edge XANES spectra and **b** Fourier-transform EXAFS spectra of Fe–N–C, Fe–N–C/control, Fe–N–C/N, Fe–N–C/FeN, Fe foil, and Fe_2_O_3_

The information on the valence state and coordination environment of Fe centers was examined by XAS. The X-ray absorption near-edge structure (XANES) of Fe K-edge spectra reveals that the Fe-K edge energies of Fe–N–C/control, Fe–N–C/N and Fe–N–C/FeN are all between those of Fe foil (Fe^0^) and Fe_2_O_3_ (Fe^3+^) (Fig. 4a), indicating a low oxidation valence of Fe single atoms. The Fourier-transformed (FT) k^3^-weighted extended X-ray absorption fine structure (EXAFS) shows that all Fe–N–C catalysts exhibit only a prominent peak at 1.5 Å (Fig. 4b), attributed to the Fe–N(O) coordination shell. There are no any other bonds, such as Fe–Fe bonds can be observed in the EXAFS, indicating the atomically dispersed feature. The structural parameters from EXAFS fitting reveal the weighted average of Fe–N coordination number for Fe–N–C/control, Fe–N–C/N, and Fe–N–C/FeN are 4.7, 4.9, and 5.4 (Table S6), respectively. It is suggested that a square-pyramid-like Fe–N_5_ configuration with a fifth axial ligand is dominated in the resultant catalysts, which is different from Fe–N–C with a coordination number of about 2.1 [43].

### Electrochemical Measurements

**Fig. 5:**
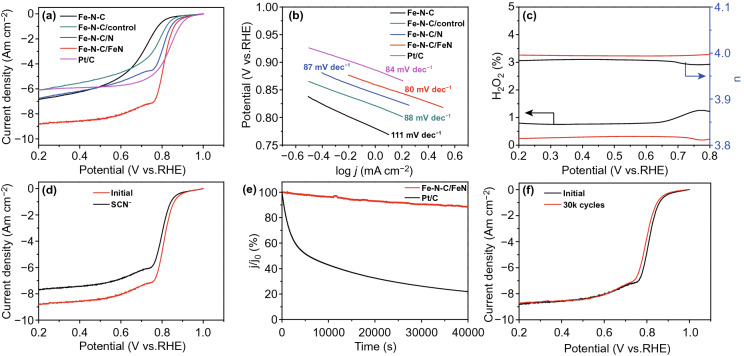
**a** LSV curves and **b** Tafel slope curves of Fe–N–C, Fe–N–C/control, Fe–N–C/N, Fe–N–C/FeN and commercial Pt/C at 1600 rpm in O_2_-saturated 0.1 M HClO_4_ solution (sweep rate: 10 mV s^−1^). **c** H_2_O_2_ yield and electron transfer number of Fe–N–C/FeN (black) and commercial Pt/C (red). **d** LSV curves of Fe–N–C/FeN obtained without and with 5 mM KSCN. **e** Chronoamperometric response of Fe–N–C/FeN and commercial Pt/C. **f** ORR polarization plots of Fe–N–C/FeN before and after 30,000 cycles

The linear sweep voltammetry (LSV) curves reveal that Fe–N–C/FeN possesses an excellent electrocatalytic activity with an *E*_onset_ of 0.96 V and *E*_1/2_ of 0.81 V (Fig. 5a), which are superior to those of Fe–N–C/N (0.96 and 0.80 V), Fe–N–C/control (0.93 and 0.78 V), Fe–N–C (0.93 and 0.72 V) and comparable to that of commercial Pt/C (0.96 and 0.83 V) (Table S7). Tafel plots of those catalysts derived from their polarization curves. As revealed in Fig. 5b, Fe–N–C/FeN possesses the smallest Tafel slope (80 mV dec^−1^), manifesting the fast kinetic of Fe–N–C/FeN for ORR process. To corroborate the superior activity of Fe–N–C/FeN, the kinetic current density of Fe–N–C/FeN (11.24 mA cm^−2^) is higher than those of Fe–N–C/N (5.85 mA cm^−2^), Fe–N–C/control (2.40 mA cm^−2^) and Fe–N–C (0.73 mA cm^−2^) via K-L equation (Table S7). As shown in Fig. 5c, electron transfer number (n) of Fe–N–C/FeN is about 4.0 with a low H_2_O_2_ yield of below 1% over the entire potential range, which is close to those of commercial Pt/C, revealing a desirable 4e^−^ pathway. To further confirm the role of FeN_*x*_, the SCN^−^ poison experiment was also carried out to block the Fe–N sites (Fig. 5d). After the introduction of SCN^−^, the *E*_1/2_ of Fe–N–C/FeN is found to shift negatively and diffusion-limiting current density is also decreased, further manifesting that the FeN_*x*_ is the active site for ORR. To investigate the methanol tolerance property and electrochemical stability, the chronoamperometry test was performed. After the addition of 1.0 M methanol, the current line of commercial Pt/C suffers the obvious change (Fig. S2). In contrast, the current of Fe–N–C/FeN remains nearly constant, which indicates that the resultant catalyst possesses strong tolerance to methanol. In the long-term stability test by chronoamperometric, 90% of the initial ORR activity was retained, which is better than that of commercial Pt/C under the same period time (Fig. 5e). Notably, the *E*_1/2_ of Fe–N–C/FeN is only observed with a slightly negative shift (19 mV) compared with initial ORR activities of Fe–N–C/FeN after 30,000 cycles (Fig. 5f), indicating the best ORR stability. Compared with the other reported NPMCs in acidic media, Fe–N–C/FeN displays the excellent electrochemical activity (Table S8).

Recent density functional theory (DFT) calculations showed FeN_5_ moiety in acidic media has a lower reaction energy barrier than other low coordinated FeN_*x*_ moieties [50, 52]. The lower energy barrier indicates good ORR activities because it makes O_2_ easier to be reduced to H_2_O. In addition, the adsorption energy of OH also has a contribution to improve the electrocatalytic activity [53, 54]. According to the previous reports, DFT calculations indicated that FeN_5_ sites have a lower reaction energy barrier (0.67 eV) and adsorption energy of intermediate OH (2.88 eV) compared with FeN_4_ sites (0.75 and 3.07 eV) or FeN_2_ sites (1.99 and 4.38 eV), facilitating the ORR activity in acid media [50]. It is believed that FeN_5_ sites have more effective ORR activity than FeN_4_ and FeN_2_ sites via DFT calculation. Experimental results and DFT calculations manifest that the outstanding ORR performance of the catalyst is significantly correlated with tuning the density and coordination environment of active sites.

## Conclusions

In summary, a secondary-atom-doped strategy was manipulated to fabricate atomical Fe sites dispersed in carbon support catalysts. Besides the optimization of microstructures and hydrophobic/hydrophilic properties of catalysts, the density and coordination environment of Fe sites in Fe–N–C/FeN catalyst also were optimized simultaneously during the secondary doping process. The obtained Fe-N-C/FeN showed comparable activity and high long-term stability for ORR relative to Pt/C in acidic electrolytes. Experimental results further confirmed that FeN_5_ sites have more effective ORR activity than FeN_2_ sites, in agreement with the DFT calculations. This work opens a new insight into rational design of robust SACs with enhanced intrinsic activity and high density of active sites, holding great promise in energy-related small-molecule electrocatalysis.

## Electronic supplementary material

Below is the link to the electronic supplementary material.

Supplementary material 1 (PDF 435 kb)
