# Supplementary material for: Secondary-Atom-Doping Enables Robust Fe–N–C Single-Atom Catalysts with Enhanced Oxygen Reduction Reaction
Source: Nanomicro Lett. 2020 Aug 12;12:163. doi: 10.1007/s40820-020-00502-5 (PMC7770947; doi:10.1007/s40820-020-00502-5)
Supplement: Supplementary file 1 — Supplementary material 1 (PDF 435 kb) [file 40820_2020_502_MOESM1_ESM.pdf]

Supporting Information for

Secondary-Atom-Doping Enables Robust Fe-N-C Single-Atom Catalysts with Enhanced Oxygen Reduction Reaction

Xin Luo[1], Xiaoqian Wei[1], Hengjia Wang[1], Wenling Gu[1], Takuma Kaneko[2], Yusuke Yoshida[2], Xiao Zhao[2,*], Chengzhou Zhu[1,*]

[1]Key Laboratory of Pesticide and Chemical Biology of Ministry of Education, International Joint Research Center for Intelligent Biosensing Technology and Health, College of Chemistry, Central China Normal University, Wuhan 430079, People's Republic of China

[2]Innovation Research Center for Fuel Cells, The University of Electro-Communications, Chofugaoka, Chofu, Tokyo 182-8585, Japan

*Corresponding authors. E-mail: xiaozhao@uec.ac.jp (Xiao Zhao); czzhu@mail.ccnu.edu.cn (Chengzhou Zhu)

Supplementary Figures and Tables

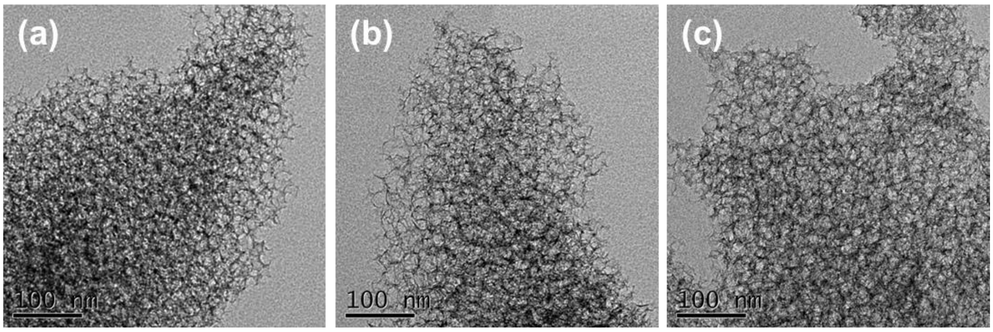

Fig. S1 a TEM images of Fe-N-C, **b** Fe-N-C/control and **c** Fe-N-C/N

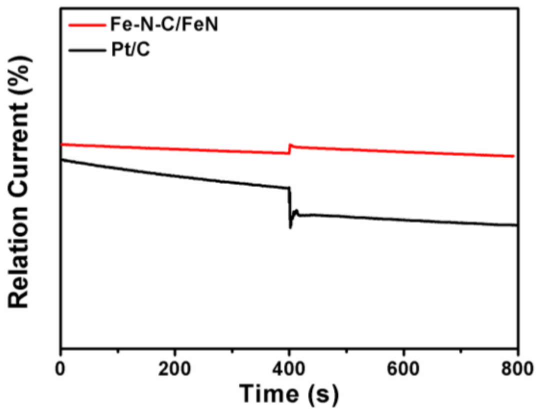

Fig. S2 Tolerance to methanol of Fe-N-C/FeN compared with Pt/C

Table S1 BET surface areas and pore distribution of different as-prepared catalysts

Catalysts	micropore volume (cm^3 g^{-1})	Smicropore-BET (m^2 g^{-1})	mesopore volume (cm^3 g^{-1})	Smesopore-BET (m^2 g^{-1})
Fe-N-C	0.11	270.5	1.6	411.2
Fe-N-C/control	0.16	364.5	2.2	579.6
Fe-N-C/N	0.14	323.3	2.1	522.0
Fe-N-C/FeN	0.09	223.9	2.0	558.2

Table S2 The N content of Fe-N-C, Fe-N-C/control, Fe-N-C/N and Fe-N-C/FeN determined by XPS

Catalysts	N (at%)	C (at%)	O (at%)
Fe-N-C	5.93	86.62	6.95
Fe-N-C/control	4.5	89.6	5.61
Fe-N-C/N	4.54	89.7	5.51
Fe-N-C/FeN	4.29	89.7	5.68

Table S3 Fitting results of C 1s XPS spectra for Fe-N-C, Fe-N-C/control, Fe-N-C/N and Fe-N-C/FeN

catalysts	C-C
Fe-N-C	34.8
Fe-N-C/control	43.3
Fe-N-C/N	43.4
Fe-N-C/FeN	44.7

Table S4 Fitting results of N 1s XPS spectra for Fe-N-C, Fe-N-C/control, Fe-N-C/N and Fe-N-C/FeN

catalysts	pyridinic N	FeN_x	Pyrrolic N	graphitic N	oxidized N
Fe-N-C	13.8	4.3	3.5	66.4	12.0
Fe-N-C/control	14.3	4.7	3.4	65.8	11.8
Fe-N-C/N	19.6	4.9	2.4	61.7	11.4
Fe-N-C/FeN	16.2	7.6	1.5	64	10.7

Table S5 Fe content in various catalysts determined by ICP-MS

catalysts	Fe (wt%)
Fe-N-C	0.19
Fe-N-C/control	0.19
Fe-N-C/N	0.135
Fe-N-C/FeN	0.38

Table S6 EXAFS fitting results for Fe-N-C based catalysts. CN: Coordination number; R: phase-corrected bond distance; σ^2: the Debye-Waller factor; ΔE_0 edge-energy shift relative to the ideal absorption edge-energy; R-factor: reliability factor

Catalysts	Fe-N(O) scattering			ΔE_0 (eV)	R-factor (%)
	CN	R (Å)	σ^2 (Å^2)		
Fe-N-C/control	4.7	1.99±0.02	0.011 ± 0.003	4.4	0.10
Fe-N-C/N	4.9	1.98±0.02	0.012± 0.003	3.3	0.07
Fe-N-C/FeN	5.4	2.00±0.02	0.013 ± 0.003	5.0	0.08

Table S7 The ORR performances of Fe-N-C, Fe-N-C/control, Fe-N-C/N, Fe-N-C/FeN and commercial Pt/C in acidic electrolytes. (E_{onset}, $E_{1/2}$, J and J_K are the onset potential at 0.1 mA cm^{-2}, half-wave potential, current density at 0.5 V and kinetic current density at 0.8 V, respectively)

Catalyst	E_{onset} (V)	$E_{1/2}$ (V)	J (mA cm^{-2})	J_K (mA cm^{-2})
Fe-N-C	0.93	0.72	5.80	0.73
Fe-N-C/control	0.93	0.78	5.18	2.40
Fe-N-C/N	0.96	0.80	5.85	5.85
Fe-N-C/FeN	0.96	0.81	8.38	11.24
commercial Pt/C	0.96	0.83	5.85	12.74

Table S8 The ORR performance of catalysts in acidic electrolyte. ($E_{1/2}$ is the half-wave potential of the catalyst; ΔE is the ORR half-wave potential shift after ADT)

Catalyst	Catalyst loading (mg cm^{-2})	E_{onset} (V)	$E_{1/2}$ (V)	References
Fe-N-C/FeN	1	0.96	0.81	This work
Fe-ISAs/CN	0.4	--	0.77	[S1]
ZIF'-FA-CNT-p	0.5	0.95	0.81	[S2]
Fe-N/C	0.8	--	0.79	[S3]
(Fe,Co)/N-C	1.1	1.06	0.86	[S4]
Fe–N/CNT	0.8	--	0.78	[S5]
Fe-NC SAC	0.6	0.98	0.68	[S6]
PANI-Fe-C	0.6	0.93	0.81	[S7]
(CM+PANI)-Fe-C	0.6	--	0.80	[S8]

Supplementary References

[S1] Y. Chen, S. Ji, Y. Wang, J. Dong, W. Chen et al., Isolated single iron atoms anchored on N-doped porous carbon as an efficient electrocatalyst for the oxygen reduction reaction. Angew. Chem. Int. Ed. **56**(24), 6937-6941 (2017). https://doi.org/10.1002/anie.201702473

[S2]C. Zhang, Y.C. Wang, B. An, R. Huang, C. Wang, Z. Zhou, W. Lin, Networking pyrolyzed zeolitic imidazolate frameworks by carbon nanotubes improves conductivity and enhances oxygen-reduction performance in polymer-electrolyte membrane fuel cells. Adv. Mater. **29**(4), 1604556 (2016). https://doi.org/10.1002/adma.201604556

[S3]Y.J. Sa, D.J. Seo, J. Woo, J.T. Lim, J.Y. Cheon et al., A general approach to preferential formation of active Fe-N_x sites in Fe-N/C electrocatalysts for efficient oxygen reduction reaction. J. Am. Chem. Soc. **138**(45), 15046-15056 (2016). https://doi.org/10.1021/jacs.6b09470

[S4]J. Wang, Z. Huang, W. Liu, C. Chang, H. Tang et al., Design of N-coordinated dual-metal sites: a stable and active pt-free catalyst for acidic oxygen reduction reaction. J. Am. Chem. Soc. **139**(48), 17281-17284 (2017). https://doi.org/10.1021/jacs.7b10385

[S5]D. Xia, X. Yang, L. Xie, Y. Wei, W. Jiang et al., Direct growth of carbon nanotubes doped with single atomic Fe–N_4 active sites and neighboring graphitic nitrogen for efficient and stable oxygen reduction electrocatalysis. Adv. Func. Mater. **29**(49), 1906174 (2019). https://doi.org/10.1002/adfm.201906174

[S6]L. Zhao, Y. Zhang, L.B. Huang, X.Z. Liu, Q.H. Zhang et al., Cascade anchoring strategy for general mass production of high-loading single-atomic metal-nitrogen catalysts. Nat. Commun. **10**, 1278 (2019). https://doi.org/10.1038/s41467-019-09290-y

[S7]G. Wu, K.L. More, C.M. Johnston, P. Zelenay, High-performance electrocatalysts for oxygen reduction derived from polyaniline, iron, and cobalt. Science **332**(6028), 443-447 (2011). https://doi.org/10.1126/science.1200832

[S8]H.T. Chung, D.A. Cullen, D. Higgins, B.T. Sneed, E.F. Holby, K.L. More, P. Zelenay, Direct atomic-level insight into the active sites of a high-performance PGM-free ORR catalyst. Science **357**(6350), 479-484 (2017). https://doi.org/10.1126/science.aan2255.
